# Rethinking Urban Water Management Through Drivers-Pressures-States-Impacts-Responses Framework Application in Chennai, India

**DOI:** 10.1007/s00267-024-02022-z

**Published:** 2024-08-06

**Authors:** Daniel Rosado, Valeria Fárez-Román, Felix Müller, Indumathi Nambi, Nicola Fohrer

**Affiliations:** 1 Department of Hydrology and Water Resources Management, Institute for Natural Resource Conservation, Kiel University, Kiel, Germany; 2 Department of Civil Engineering, Indian Institute of Technology Madras, Chennai, India; 3 Indo-German Centre for Sustainability, Indian Institute of Technology Madras, Chennai, India; 4 Department Lake Research, UFZ - Helmholtz Centre for Environmental Research, Magdeburg, Germany; 5 Department of Ecosystem Management, Institute for Natural Resource Conservation, Kiel University, Kiel, Germany

**Keywords:** Water Resources Management, Land use change, Water scarcity, Environmental causal framework, Urban resilience, South India

## Abstract

Cities suffering water scarcity are projected to increase in the following decades. However, the application of standardized indicator frameworks for assessing urban water resource management problems is on an early stage. India is expected to have the highest urban population facing water scarcity in the world by 2050. In this study, the authors assess how the Drivers-Pressures-States-Impacts-Responses framework, a causal framework adopted by the European Environment Agency, can contribute to evaluate water management challenges in cities and apply it to Chennai, India´s fourth-largest urban agglomeration. The framework proved to be a helpful tool for the evaluation of water management challenges in cities by disentangling relationships between environmental indicators and structuring dispersed data that allows a better understanding for policymakers. The main drivers identified in Chennai were population growth and economic development which generated impacts such as loss of aquatic ecosystems, low water table, low water quality, and reduction of biodiversity and human health. As a response, better urban planning, projects for new water infrastructure, and water bodies restoration have been implemented. Nevertheless, Chennai keeps facing difficulties to achieve proper water management. The severe hit of the COVID-19 pandemic on the Indian economy and its future management will be key for achievements related to water management.

## Introduction

Cities around the globe are suffering from water scarcity. In 2009, at the end of the “Millennium Drought”, water storage volumes feeding Melbourne fell to a historic low of 25.6% of capacity (Low et al. 2015; van Leeuwen 2017). In 2013 and 2014, Sao Paulo became dependent on reservoirs with available water percentages in the single digits (Millington 2018). In 2018, Cape Town faced a Day Zero in which pipes went dry and people needed to collect drinking water from distribution points (Zetland 2021).

Urban water demand will increase by 80% by 2050, while rapid population growth and urbanization of cities put significant pressure on water resources (Newton 2016) and climate change is altering the timing and distribution of water (Flörke et al. 2018). In this context, the number of large cities facing water scarcity was projected to increase from 193 (37%) to 292 (56%), and the global urban population facing water scarcity was projected to double from 933 million (33%) in 2016 to 1.693–2.373 billion (35–51%) in 2050 (He et al. 2021). Consequently, water insecurity will be one of the most severe crisis the world will face during the next several decades (UNESCO and UN-Water 2020). Cities are also facing a problem of water quality with impacts on health and ecosystems (Chaturvedi 2012). In 2020, at least 2 billion people used a drinking water source contaminated with feces (WHO World Health Organization 2020), and 3.6 billion lacked safely managed sanitation (World Bank 2020).

India had the highest number of urban inhabitants facing water scarcity worldwide in 2016 (222 million people). This is expected to increase to 550 million in 2050, i.e. 26.7% of the world’s urban population facing water scarcity (He et al. 2021). City development and poor institutional control (Chaturvedi 2012; Hossain et al. 2013) have resulted in the pollution of the majority of surface water bodies with untreated sewage (Chaturvedi 2012) and promoted over-extraction of groundwater (Wada et al. 2012). Chennai, India´s fourth-largest urban agglomeration, exhibits a complex setup around water resources management that generates social, political, and environmental tensions, including water-poor areas where human development is limited (Allen et al. 2006). In 2019, the city hit Day Zero when its four major reservoirs dried up and four million people were relying solely on water tankers (Jayaraman 2019; “Water crisis in Chennai”, 2019).

Traditionally, cities relied on large-scale infrastructure for water management, often with systems operating independently of each other (Brown et al. 2009; Marlow et al. 2013). Addressing water management in cities with complex scenarios like Chennai requires an integrated approach, such as the Integrated Urban Water Management (IUWM) process. This approach encompasses water supply, sanitation, stormwater, and wastewater management integrating them with land use planning and economic development (Bahri 2012). The concept of IUWM emerged in the 1980s, highlighting the interconnections between actors, surface water, groundwater, and issues of water quantity and quality, and has evolved over the years (Benson et al. 2015; Hoekstra et al. 2018; Manny 2022). In the 1990s, Sustainable Urban Water Management (SUWM) became prominent, driven by growing concerns for ecological health and sustainable development, integrating environmental variables more comprehensively into the discussion (Hoekstra et al. 2018; Pluchinotta et al. 2021). In the late 2000s, Adaptive Water Management emerged in response to the need for climate change adaptation (Kirono et al. 2014; Hoekstra et al. 2018). Recently, concepts such as Water Risk, Water Resilience, the Water-Food-Energy Nexus, Total Water Cycle Management (Maurya and Singh 2022), and Water Sensitive Urban Design (van der Meulen et al. 2023) have become influential (Hoekstra et al. 2018). In parallel and since around 2000, water security has also emerged as a central concept, broadly encompassing integrated, sustainable, and adaptive aspects (Hoekstra et al. 2018). The relevance of water security is underscored by its recognition and definitions from the Global Water Partnership (GWP 2000), the World Water Council (WWC 2000), and the United Nations (UN-Water 2013). Over time, the term has evolved to include various attributes, such as affordability, human well-being, water-related disasters, hazard, risk, political stability, and resilience (Romero-Lankao and Gnatz 2016; Chapagain et al. 2022).

Although IUWM is a crucial process to prevent water crises regardless of water scarcity (Di Baldassarre et al. 2018) and has the highest potential to reduce pressures on water resources (Koop and van Leeuwen 2015a), there is no internationally standardized indicator framework for it (Koop and van Leeuwen 2015a). This situation compromises the achievement of the United Nations Sustainable Development Goals (SDGs) numbers 6 Clean Water and Sanitation and 11 Sustainable Cities and Communities (United Nations 2015; He et al. 2021).

Several models and frameworks are used for analyzing water management, environmental issues, and socio-ecological systems: causal-chain frameworks, such as the Pressure-State-Response (PSR), Pressure-State-Impact-Response (PSIR), and the Drivers-Pressures-States-Impacts-Responses (DPSIR), sustainability assessment frameworks, including the Social-Ecological Systems Framework (SESF) and the Ecosystem Services Framework, and integrated water management frameworks, like the City Blueprint and the Asian Water Development Outlook (AWDO) frameworks.

The PSR framework emphasizes the relationship between environmental pressures, the current state of the environment, and societal responses (Maurya and Singh 2022), while the PSIR framework incorporates an “Impact” stage (Van Ginkel et al. 2018). The DPSIR, a causal framework for describing the interactions between society and the environment, provides an organized structure to assessing the causes, consequences, and responses to changes in ecosystems (Gabrielsen and Bosch 2003; European Environment Agency 2020). The SESF is used to diagnose the sustainability of social-ecological systems (Ostrom 2009), while the Ecosystem Services Framework categorizes the benefits humans obtain from ecosystems, with an emphasis on natural resource management (Matzdorf & Meyer, 2014). The City Blueprint framework (van Leeuwen et al. 2012; Koop and van Leeuwen 2015b) uses 24 indicators across eight categories, including water security, water quality, sanitation, biodiversity, and governance. It measures integrated water resources management performance implemented by local water authorities (Hoekstra et al. 2018) and includes an additional framework for describing significant trends and pressures that may impact water management (Koop and van Leeuwen 2015b). The AWDO framework (Asian Development Bank 2020) assesses water security through five key dimensions: households, economies, cities, the environment, and resilient communities.

The DPSIR framework is particularly suitable for urban water management due to its holistic and integrated approach. Simplified frameworks like PSR and PSIR are straightforward, easy to use, and effectively link pressures to state changes and policy responses (Hazbavi et al. 2020). This clarity makes them useful for decision-makers. However, they often lack the depth required for comprehensive urban water management by oversimplifying interactions (Niemeijer and De Groot 2008; Levrel et al. 2009). Compared to SESF and the Ecosystem Services Framework, DPSIR not only addresses sustainability and ecosystem services but also analyzes the system with a holistic approach, which is key for complex scenarios like water management in Chennai. The City Blueprint framework provides a detailed structure but is complex, requiring a dual framework that focuses on water resources management performance as well as trends and pressures. This complexity, along with the need for extensive and specific data, can impede its application, especially in developing countries (Hoekstra et al. 2018). The AWDO framework provides a holistic view of water security by addressing interconnected and interdependent dimensions. However, it is primarily designed for national assessments, includes only five urban-specific indicators, and uses averaged data for all urban areas across a country, which limits its usefulness for IUWM (Jensen and Wu 2018).

Adopted by the European Environment Agency, the DPSIR framework provides an organized structure to assess the causes, consequences, and responses regarding water management (European Environment Agency 2020), that facilitates communication with stakeholders (Tscherning et al., 2012). Additionally, it has been widely applied to assess the environmental state of cities (Kohsaka 2010), rural communities (Gari et al. 2018a), and river basins (Kong et al. 2016; Pagan et al. 2020a), and has demonstrated its utility to describe cause-effect relationships in cases with limited data availability (Gari et al. 2018b), and to develop causal chains (Pagan et al. 2020b).

Hence, this study aims to address the following research questions: (i) How can the DPSIR framework contribute to evaluate water management challenges in cities with wicked water problems? (ii) What are common limitations for effective water management in cities located in developing countries and facing resource scarcity? These questions will be addressed through the application of the DPSIR framework to the city of Chennai, India.

## Materials and Methods

### Study Area

**Fig. 1:**
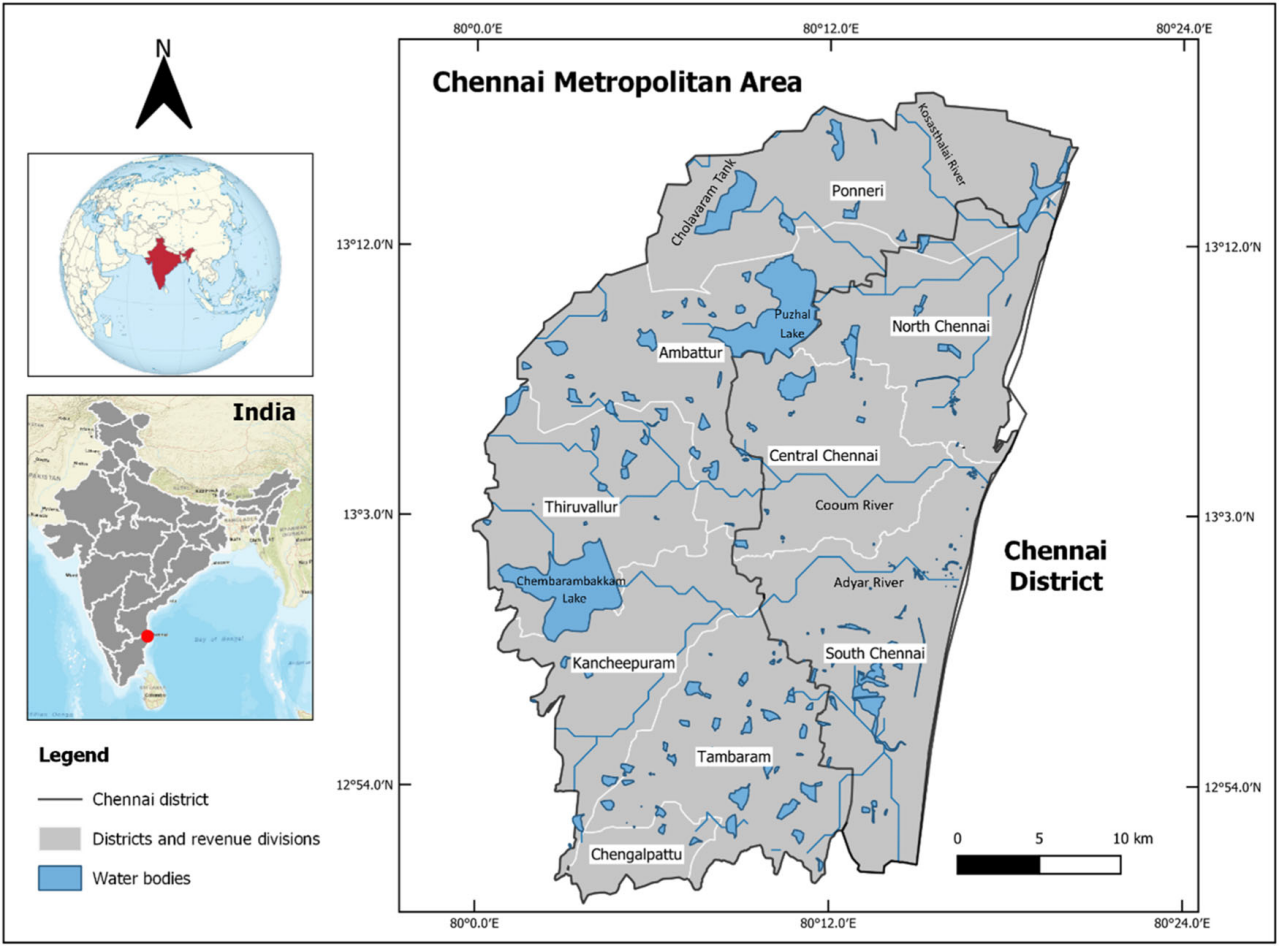
Chennai location and water bodies. Source: Authors. Note: India on the globe image, Wikimedia Commons, 2014 (https://creativecommons.org/licenses/by-sa/3.0). CC BY-SA 3.0

Chennai, known as Madras until 1996, is the capital of the Indian state of Tamil Nadu. It is located on the southeastern coast of India (13^o^05′N and 80^o^18′E), on the Bay of Bengal (Fig. 1). Nowadays, the Chennai city limits coincide with the Chennai district (Government of Tamil Nadu 2020). According to the 2011 Indian census, the Chennai district had 7,088,000 inhabitants in an area of 426 km^2^ (Directorate of Census Operations Tamil Nadu 2011). Chennai city is governed by the Greater Chennai Corporation (Chennai District 2020).

The Chennai city district together with parts of Tiruvallur, Kanchipuram, and Chengalpattu districts constitute the Chennai Metropolitan Area (CMA) with an extension of 1189 km^2^ (CMDA 2020) and 8.70 million inhabitants according to the 2011 Indian census (Directorate of Census Operations Tamil Nadu 2011). The Chennai Metropolitan Development Authority is in charge of urban planning in this area.

Chennai has a tropical wet and dry climate (Köppen: Aw) with a relative constant temperature (Rajanikanth and Rajini Kanth 2020). In the period 1971–2000, the highest and lowest average monthly temperatures showed annual variations ranging from 28.9 to 37.1 °C and from 21.2 to 28.0 °C, and the average monthly rainfall ranged from 2.2 to 407.4 mm (Selvaraj et al. 2016). The hottest part of the year is late May and early June and the coolest is January (Prakash and Punyaseshudu 2015). The average annual precipitation is around 1,400 mm (Rajanikanth and Rajini Kanth 2020). The lowest precipitations occurred in February, during the pre-monsoon season, and the highest from mid–October to mid–December in the northeast monsoon (NE) season (Selvaraj et al. 2016). Regarding climate change, mean temperatures have risen (maximum, 1.6 °C; annual, 1.3 °C; minimum, 1.0 °C) from 1951 to 2010 (Jeganathan and Andimuthu 2013) while rainfall tendency is unclear (Ramachandran and Anushiya 2015; Rangarajan et al. 2018). Concerning floods, Oldenborgh et al. (2016) found no signal for a positive trend in extreme one-day precipitation at the southeastern coast of India over 1900–2014.

Chennai is situated on a flat area known as the Eastern Coastal Plains with an average elevation of 6.7 m.a.s.l. and a highest point of 60 m.a.s.l. (Pulikesi et al. 2006). The rivers of Chennai flow from west to east, draining into the Bay of Bengal, and divide the city into north–south sections. Two major rivers are the Cooum River, located in the center of the city, and the Adyar River to the south. A third river, the Kortalaiyar, flows through the northern fringes of the city before draining into the sea, at Ennore. The three rivers are connected by the Buckingham Canal, a freshwater artificial waterway parallel to the Coromandel Coast. Additionally, there are four reservoirs called Poondi, Cholavaram, Puzhal, and Chembarambakkam (Mariappan 2014) and many artificial lakes, locally called tanks that traditionally stored water during the monsoon season that were used for irrigation during the dry season (Devi et al. 2020).

The Chennai ecosystem has been dominated by Palmyra and Phoenix palms interspersed with thickets with a mixture of grasses and short herbs in the intervening spaces; allowing free surface water flows and preventing flooding. Trees were sparse and local (Ranjit-Daniels et al. 2020). Pallikaranai Marsh stands out with multiple species of plants, fish, birds, butterflies, and reptiles (Surya 2016) although it has been heavily urbanized within the last 30 years (Coelho 2018).

### Application of the DPSIR Framework

**Fig. 2:**
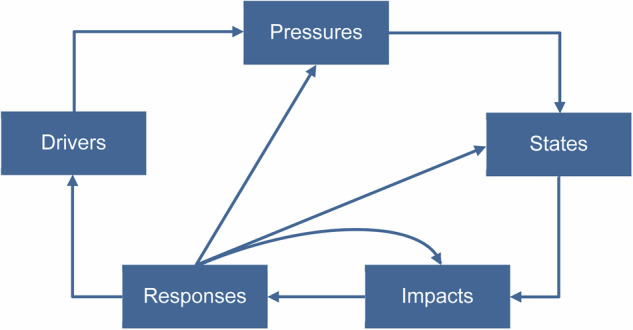
The components of the DPSIR framework and their connections

In this work, we applied the DPSIR framework (Gabrielsen and Bosch 2003; Kristensen 2004; European Environment Agency 2020) and, consequently, defined its components (drivers, pressures, states, impacts, and responses) and its causal chain in relation to water management in the city of Chennai, India. The components of the DPSIR framework and their linkages are depicted in Fig. 2 while their definitions can be found in the literature (Smeets and Weterings 1999; Gabrielsen and Bosch 2003; Kristensen 2004; Semeoshenkova et al. 2017).

The application of the DPSIR framework and the definition of its components involved extensive literature research in Scopus, Web of Sciences core collection, and Google Scholar (Patrício et al. 2016). Also, data reports and other documents provided by public administrations. Thus, the websites of the Chennai Metropolitan Water Supply and Sewerage Board (CMWSSB, also known as Metrowater), Chennai Metropolitan Development Authority (CMDA), Tamil Nadu Water Supply and Drainage (TWAD) Board, Tamil Nadu Pollution Control Board, Central Pollution Control Board, Central Ground Water Board (CGWB) were consulted, as well as the second edition of the master plan of Chennai and renowned newspapers.

## Results

### Overview

**Fig. 3:**
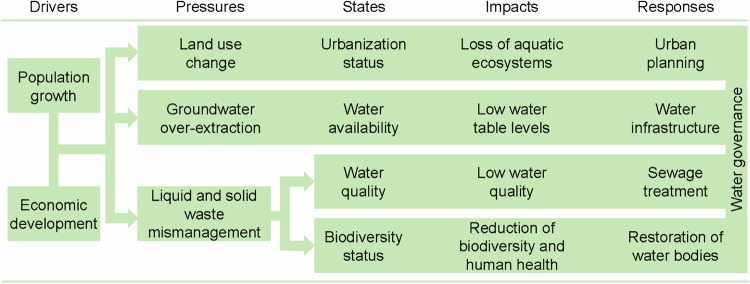
Components and causal chain of DPSIR analysis of the water management in Chennai, India

Figure 3 presents the components and causal chain of DPSIR analysis of water management in Chennai. Also, a table shows the indicators used in this study in the supplementary material (Table S1).

### Drivers

#### Population Growth

**Fig. 4:**
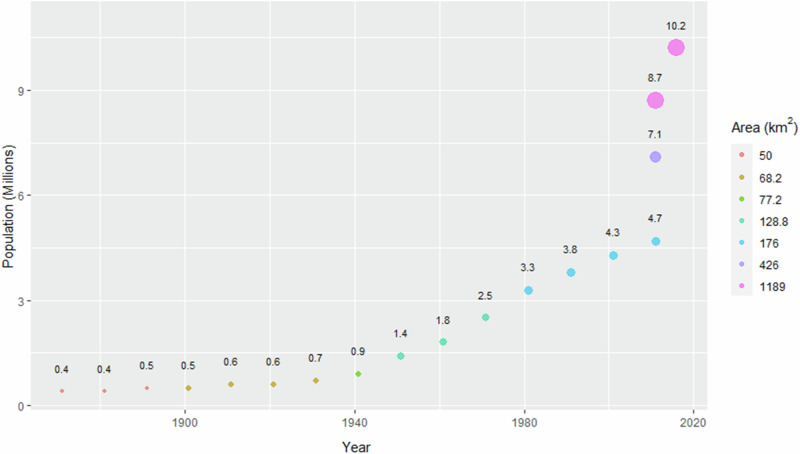
Population and area of Chennai City (1871–2011) and Chennai Metropolitan Area (2011–2016). Sources: 1871–1891, Secretary of State for India (1908); 1901–2001, CMDA (2008); 2011, Directorate of Census Operations Tamil Nadu (2011); 2016, United Nations (2016). Populations of 2011 from bottom to top correspond to (i) the area before the expansion of the Chennai City District in 2011, (ii) the area after the expansion, and (iii) the metropolitan area

Chennai is today a cosmopolitan megacity due to a transformation that started in the last century and continues in the present. The 20th century brought to Chennai an accelerated population growth rate over a decadal 10% (Fig. 4). During the period 2001–2011, the population growth rate equaled 7.8% due to the almost complete urbanization within the city limits of that time (174 km^2^), an average annual growth of 0.75%. Later in 2011, the area of the city was expanded to 426 km^2^ and its population reached 7,088,000 (Directorate of Census Operations Tamil Nadu 2011). Considering the new limits, the growth rate in the period 2001–2011 was above 10%. Since then, the Chennai city limits remain untouched and coincide with the Chennai district, one of the 38 districts of the State of Tamil Nadu.

In parallel to the population, the spatial area of Chennai has expanded from 68 km^2^ to 426 km^2^ since 1901 (Krishnamurthy and Desouza 2015), and the population density has increased from 1041 (1981) to 2109 (2011) persons per km^2^ (Aithal and Ramachandra 2016).

The Chennai Metropolitan Area is composed by the Chennai District and parts of Tiruvallur, Kanchipuram, and Chengalpattu districts, the latter carved out of Kanchipuram District in November 2019 (CMDA 2020). It has an area of 1189 km^2^ and 8.70 million inhabitants according to the 2011 Indian census, the fourth most populated metropolitan agglomeration in India (Directorate of Census Operations Tamil Nadu 2011). Additionally, its population has been estimated to be 10.2 million in 2016 (30th-largest in the world) and to reach 13.9 million in 2030 (United Nations 2016).

The constant immigration plays a key role for the population growth of Chennai, with a migrant population of 918,000 (23.9% over the total) in 1991, and 937,000 (21.57%) in 2001. In the latter, divided into 74.5% from other parts of Tamil Nadu, 23.8% from other parts of India, and 1.7% from foreign countries (CMDA 2008). Since 2000, the population from foreign countries has increased from 35,000 in 2009 to 82,790 in 2011 (Krishnamurthy and Desouza 2015). Traditionally, the high fertility rate also explained the population growth. However, today in Tamil Nadu it is among the lowest of the country and it is similar to developed countries decreasing from 2.5 in 1993 to 1.7 in 2016 (International Institute for Population Sciences 2017; NITI Aayog 2020). This rapid population growth increases the demand for water and the scarcity and shortages likelihood.

#### Economic Development

During the British rule of India, Chennai was the capital of the Madras Presidency and therefore the economic center of South India (Haufe 2017). Today, Chennai is one of the most economically developed and industrialized cities in India, and the largest industrial center in South India (Bloodgood 2007; Krishnamurthy and Desouza 2015).

**Fig. 5:**
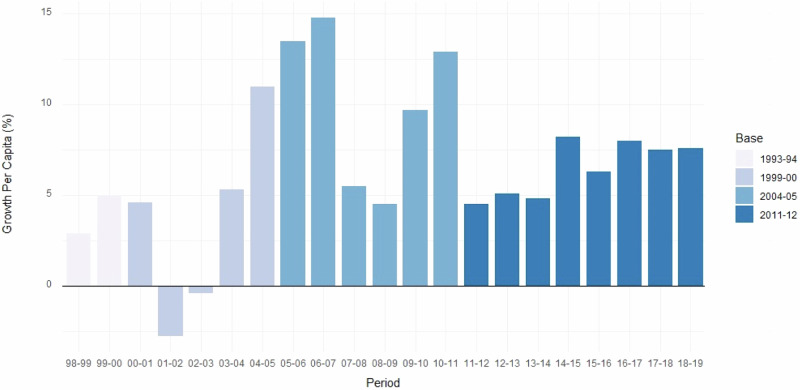
Per capita net state domestic product (NSDP) growth in percentage (rupees) at constant prices in Tamil Nadu (1998–1999 to 2019–2020). Source: Reserve Bank of India (2020). Average exchange rate (2021) is 73.9339 INR for 1 USD

Several factors have contributed to its explosive economic development. At a national scale, after the 1947–1991 protectionist period, the economy of India was liberalized and received massive foreign investments. Since the start of the 21st century, annual average gross domestic product (GDP) growth has been around 6–7% (Kanungo et al. 2018), and from 2014 to 2018, India was the world’s fastest-growing major economy with an average of 7.4%, surpassing China (International Monetary Fund 2019). At a state level, Tamil Nadu is the second largest state in India by GDP and the per capita net state domestic product in percentage at constant prices of Tamil Nadu (Fig. 5) has increased at an average 6.59% between financial years 98–99 and 18–19 (Reserve Bank of India 2020).

At a city level, Chennai received large-scale investments from multinational companies and tremendous industrialization took place, especially in the automobile, health, IT, and banking sectors (Krishnamurthy and Desouza 2015). With an estimated GDP (PPP) of $78.6 billion in 2018, Chennai metropolitan area is the fifth largest city by GDP in India (Haritas 2017). Other important industries include petrochemicals, textiles, apparels, and the ports of Chennai and Ennore. The main industrial developments of Chennai took place along three main corridors: the Old Mahabalipuram Road alongside the coast, as the IT corridor; the Grand Southern Trunk (GST) Road, towards the southwest, as the logistics and industries corridor; and the Chennai–Bengaluru Highway as an automotive and electronic hardware corridor (Haufe 2017). This economic development increases the per capita consumption of water due to new industries and the higher purchasing power of the population (Bao and Chen 2015).

### Pressures

#### Land Use Change

**Fig. 6:**
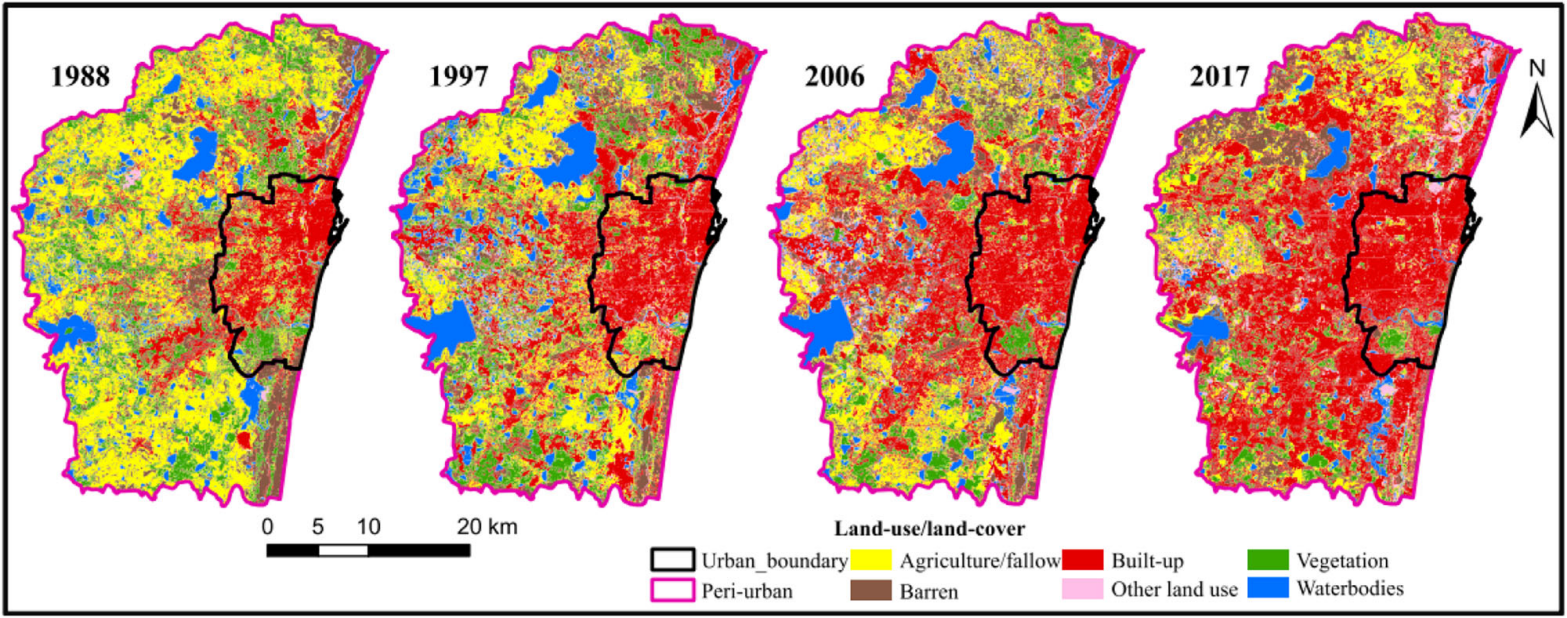
Land use change in Chennai Metropolitan Area from 1988 to 2017. Derived from LANDSAT data. Taken with permission from Mathan and Krishnaveni (2020)

Chennai experienced significant changes in land use because of intense urbanization due to rural-to-urban migration, the natural increase of population, reclassification of rural settlements into urban, and changes in boundaries of existing urban settlements. Urban areas expanded by 173.8% (211.2 km^2^ to 578.4 km^2^) in the period 1988–2017, i.e. an average annual growth of 1.92%, while rural areas and water bodies shrank as depicted in Fig. 6 (Mathan and Krishnaveni 2020).

#### Groundwater Over-extraction

The growing water demand by the increasing industries and population has led to an uncontrolled extraction of groundwater within the city and its peri-urban areas estimated on average in 200 million liters per day (Venkatachalam 2015) despite the Chennai Metropolitan Area Groundwater Act from 1987 prohibition to extract groundwater in 302 villages. This regulation is frequently violated (Butterworth et al. 2007). Many peri-urban farmers have shifted from agriculture to water production (Roumeau et al. 2015) and sell groundwater directly to Metrowater (Ruet et al. 2007) and private tankers (Butterworth et al. 2007). In the city, around 6530 deep bore wells have been dug (Venkatachalam 2015). The general status of aquifers is monitored by the public authorities (Roumeau et al. 2015).

#### Liquid and Solid Waste Mismanagement

According to Metrowater (CMWSSB 2020a), Chennai generates 550 million liters per day (MLD) that are treated in 12 sewage treatment plants with a total capacity of 727 MLD. Goutham (2017) agrees 550 MLD are treated, but also that around 1500–2000 MLD of sewage are generated in the city. Hence, nearly 1073 MLD of sewage would be directly discharged into water bodies and only 33.90% would be treated. Several areas lack access to an efficient sewage system, therefore wastewater is disposed in septic tanks (Thirumurthy 2017).

Chennai currently exhibits the highest per capita solid waste generation rate in India: 0.65 kg/person/day. Municipal Solid Waste generation has increased from 600 to 5400 metric tons per day (MTD) within 20 years with residential (68%) as main origin (Greater Chennai Corporation 2020). Collected waste is disposed with limited processing at two non-technically managed open dumpsites (Greater Chennai Corporation 2020): Kodungaiyur (1.089 km^2^) and Perungudi (0.809 km^2^). An important amount of waste remains unattended at collection centers, roadsides, drains, and riverbanks (Esakku et al. 2007; Mahajan 2016).

### States

#### Urbanization Status

Urban areas expanded by 173.8% (211.2 km^2^ to 578.4 km^2^) in the period 1988–2017 (Mathan and Krishnaveni 2020) and reached 48.7% of the Chennai Metropolitan Area surface (1189 km^2^). Chennai´s urban core is intensely developed and is surrounded by rural or peri-urban areas that lack basic amenities. Slums that lack access to water, sanitation, and electricity are home to 1.3 million people (CMDA 2006; Saharan et al. 2018) which was estimated to be 18.9% of the urban population in 2001 (CMDA 2008) and 28% in 2011 (Directorate of Census Operations Tamil Nadu 2011).

#### Water Availability

Chennai has been historically in water deficit because of the lack of perennial rivers due to the monsoon climate. The city has the lowest per capita water availability of all the metropolitan cities in India (CMDA 2006; Packialakshmibb et al. 2011). The water supply is around 90 liters per capita a day and is reduced during the dry period (Feb–Mar). Water demand for Chennai urban area in 2014 was 1173 MLD, while the supply was only 831 MLD (Government of Tamil Nadu 2014).

The core of the Chennai Metropolitan Area benefits of a better water supply (Government of Tamil Nadu 2014). In other areas, it is only 30–40 liters per capita a day, is only available on alternate days or for only 2 to 6 h per day (CMDA 2006). Around 20% of the population in slums lack of piped water and rely on public fountains and mini tanks (CMDA 2006).

Drought events are common in Chennai. Between 2003 and 2004, the entire piped water system was inoperative. The packaged water market boomed, resulting in a downward spiral of over-extraction of groundwater and unequal water distribution (Roumeau et al. 2015). During the summer of 2019, all four main reservoirs run dry after two years of scarce rainfall and 200 days without rain (Dhillon 2019; Upadhyay 2019). Tap water stopped running and costly private water tankers emerged as alternative water sources (Karthikeyan 2019) making the poor vulnerable (Masih 2019).

#### Water Quality

The numerous sources of pollution, such as sewage discharge, have reduced the quality of the water bodies (Table 1). All water bodies fail to comply in one or more parameters with the guidelines for the propagation of wildlife in surface water provided by Water Quality Standards in India (BIS 1992): pH (6.5–8.5), electrical conductivity (<1000 µS/cm), dissolved oxygen (≥4 mg/L) and BOD (≤2 mg/L). Precisely, pH is not meet in the tanks, electrical conductivity, and dissolved oxygen in Cooum River and Buckingham Canal, BOD in Adyar River, and the three water bodies mentioned previously.

**Table 1:** Values of environmental quality parameters in water bodies of Chennai, India

		In situ parameters					
Waterbody		pH	EC (µS/cm)	TDS (mg/L)	DO (mg/L)	Turbidity (NTU)	Reference
Cooum River	SW - Pr	7.3–7.9	1696–14,160	1103–9820	0–3.8		Dhamodharan et al. (2016)
	SW - Mo	7.0–7.4	1510–7140	920–4385	0.2–0.4		
	SW - Po	7.3–8.1	2260–42,400	1308–25,520	0		
	SW			1119–20,553	BDL–4.75		Nethaji Mariappan et al. (2017)
	GW	7.5–8.6	1760–2540	859–1611			
Buckingham Canal	SW	7.24–8.36	1546– 2215	1422–4250	3.5–5.9	150–258	Kumar et al. (2018)
Lakes (tanks): Porur, Perungudi, Velachery, Nandhivaram and Karapakkam	SW	6–8.7					Raji and Abraham (2018)
Groundwater City area	GW	6.0–8.2	580–7250	374–4669			Krishna Kumar et al. (2015)
Ennore creek	SW	6.2–8.1			4.5–6.1		Vasanthi et al. (2017)
Coast of Chennai	SW - Dr	7.17–8.60			0.00–12.37		Mishra et al. (2015)
	SW - Wt	7.30–8.70			0–9.98		

		Chemical and biological parameters											
Waterbody		COD (mg/L)	BOD (mg/L)	NH_3_ (mg/L)	NO_2_^−^ (mg/L)	NO_3_^−^ (mg/L)	TN (mg/L)	PO_4_^3−^ (mg/L)	TP (mg/L)	SO_4_^2−^ (mg/L)	Tot Alk (mg/L)	Chlorides (mg/L)	Reference
Adyar River	SW		20–78										Kumar et al. (2019)
Cooum River	SW - Pr	113–376	35–128	11–18.5	0–0.1	0–2.2		0.8–3.8		280–4360	194–440	308–4829	Dhamodharan et al. (2016)
	SW - Mo	102–236	35–85	9–19	BDL	BDL		0.2–2.5		140–815	176–340	140–1529	
	SW - Po	124–396	36–128	11–20	0–0.29	0–2.9		3.1–8 0–0.29		780–6660	288–549	410–10,147	
	SW	195–1204	16–375									349–990	Nethaji Mariappan et al. (2017)
	GW					0–4				117–672	165–354	284–347	
Buckingham Canal	SW	640–1572	146–511								150–1396		Kumar et al. (2018)
Lakes (tanks): Porur, Perungudi, Velachery, Nandhivaram and Karapakkam	SW	13–102	9–34				0.4–2.4		0.02–0.162				Raji and Abraham (2018)
Groundwater City area	GW					0.1–3.9				22.2–98.6	18.3–360	115–2012	Krishna Kumar et al. (2015)
Coast of Chennai	SW - Dr		0.32–148	0–3.15	0–2.37	0–7.62		0–14.02					Mishra et al. (2015)
	SW - Wt		0.01–168	0.002–2.88	0–0.749	0–4.19		0–20.02					

		Major and trace elements												
Waterbody		Ca (mg/L)	Mg (mg/L)	Na (mg/L)	K (mg/L)	Zn (mg/L) (mg/kg)	Cu (mg/L) (mg/kg)	Pb (mg/L) (mg/kg)	Cd (mg/L) (mg/kg)	Hg (mg/kg)	Fe (mg/L) (mg/kg)	Cr (mg/L) (mg/kg)	Ni (mg/L) (mg/kg)	Reference
Cooum River	SW - Pr	40–244	24–255	296–4803	21.3–136									Dhamodharan et al. (2016)
	SW - Mo	36–144	20–140	208–1402	10–75									
	SW - Po	208–593	15–43	405–9250	27–227									
	SW					0.34–2.41	0.11–0.86	BDL-0.09						Nethaji Mariappan et al. (2017)
	GW	40–68	63–107	110–357	3–9									
Pallikaranai marshland	SW					0.002–0.14	0–0.02	0.03–1.13	0–0.019		0–1.52	0.10–1.52	0–0.60	Karpagavalli et al. (2012)
	SD					149.4	102	36.6	1.6	0.58	30,201	382	85	Jayaprakash et al. (2010)
Groundwater City area	GW	26–130	1.2–141.6	71–1200	3–36									Krishna Kumar et al. (2015)
Ennore creek	PV^a^					4.658	3.289	0.761	0.416		8.957			Vasanthi et al. (2017)
	PV^b^					3.378	3.098	0.892	0.315		7.987			

Results are in surface water (SW), groundwater (GW), sediment (SD), and mussel *Perna viridis* (PV^a^: gills; PV^b^: hepatopancreas). In the major and trace elements section of the table, results in surface water and groundwater are in mg/L and results in sediments and in *Perna viridis* are in mg/kg

*Pr* Pre-monsoon, *Mo* Monsoon, *Po* Post-monsoon, *Dr* dry season, *Wt* wet season, *BDL* below detection limit

#### Biodiversity Status

Encroachment and pollution of natural and artificial water bodies have affected Chennai’s aquatic biodiversity. Amali et al. (2019) found that the wetlands located within the coastal districts of Tiruvallur, Chennai, and Kancheepuram were only 15.8% of the total area in 2016. In the Pallikaranai marshland species diversity has been reduced due to wetland occupation and wastewater and solid waste pollution. According to Azeez et al. (2007) and Vencatesan (2007), 114 plant and 223 animal species can be found in the wetland, including endangered species. Plants include two endemics to Peninsular India (*Cynodon barberi* and *Iseilema enthephroides*), and exotic plants like water hyacinth (*Eichhornia crassipes*) and water lettuce (*Pistia stratiotes*). 115 bird, 46 fish, 21 reptile, 10 amphibian, 10 mammal, 9 mollusk, 5 crustacean, and 7 butterfly species have been reported. Raj et al. (2010) reported 101 species of resident and migratory birds. Knight and Devi (2010) found 75 fish species on the freshwater habitats around Chennai.

### Impacts

#### Loss of Aquatic Ecosystems

Urbanization involved occupation, mismanagement, and drainage of wetlands, and the abandonment of water tanks (Amirtham et al. 2009; Meenatchi Sundaram 2011; Murawski 2015). Therefore, water bodies shrank by 30.6%, i.e. an average annual reduction of 1.25% (Mathan and Krishnaveni 2020).

The Pallikaranai marshland drained about 250 km^2^ and occupied an area of 56 km^2^ over today’s residential localities such as Velachery, Thoraipakkam, Perungudi, and Pallikaranai until 1980 (Vencatesan 2007; Sree Sharmila and Swathika 2016). Construction in this area reduced its extension to only 6 km^2^ (Steinbruch and Hörmann 2015; Sree Sharmila and Swathika 2016). In 2005, 0.57 km^2^ was used as a dumpsite and 1.37 km^2^ was impacted by garbage and sewage (Vencatesan 2007). In 2007, 3.17 km^2^ was declared as a Reserve Forest (Vencatesan 2007). Nowadays, there are two garbage-dumping yards in its outskirts. Into the bargain, untreated or partially treated sewage enters the wetland due to direct discharge or the Perungudi sewage treatment plant (The Hindu 2019).

Shifting the drinking water supply from surface water to groundwater sources in the past resulted in the degradation and abandonment of water tanks (Ariza et al. 2007; Palanisami et al. 2008). The Tamil Nadu administration tried to change this situation with the Protection of Tanks and an Eviction of Encroachment Act, in October 2007. However, this law has not been fully implemented (Lavanya 2013). Approximately 15% of the water storage capacity of the tanks in the Adyar basin was lost due to heavy siltation (Massuel et al. 2014).

#### Low Water Table

Groundwater over-extraction and reduced groundwater recharge due to dense urbanization and the increase of paved areas have caused declining groundwater levels (Srinivasan et al. 2013). Subsequently, aquifers are salinized due to seawater intrusion (Butterworth et al. 2007; Brunner et al. 2014; John 2015).

According to the Central Groundwater Board, 80% of Chennai’s groundwater has been depleted (Balan et al. 2012) and all of the 20 groundwater assessment units in Chennai are over-exploited. In May 2019 (pre-monsoon), 64% of 11 monitored wells presented a water level of 5–10 m and 36% of 2–5 m (Central Ground Water Board 2020). Any further exploitation could lead to severe saltwater intrusion (Balan et al. 2012). In peri-urban areas, groundwater over-extraction has caused a decline of the groundwater table: the pre-monsoon and post-monsoon groundwater level fluctuation varied from 2–6 m to 0–5 m, respectively, during 1971–2007 (Packialakshmibb et al. 2011). An evaluation of the total change in groundwater storage across the state of Tamil Nadu from 2002 to 2012 concluded that the groundwater depletion rate is 8% higher than the annual recharge rate (Chinnasamy and Agoramoorthy 2015).

#### Low Water Quality

The estimated 1073 MLD of sewage directly discharged into water bodies, together with industrial waste water produced a poor quality status of the aquatic ecosystems of Chennai (Amirthalingam 2003).

The Adyar River suffers from agricultural pollution upstream Nandambakkam, where it starts to receive untreated sewage (Venugopal et al. 2009; Lakshmi and Deepa 2011). This river is not perennial and only carries enough water during the rainy season. The rest of the year is almost stagnant due to the lack of water and the formation of sand bars near the mouth (Lakshmi 2011), transporting undiluted effluents from sewage treatment plants (CMDA 2008). The situation is similar in the Cooum River (Dhamodharan et al. 2019). Beyond Vanagram up to its mouth, the river is highly polluted due to its perennial state, siltation, sand bar formation, and almost stagnant state (Mukhopadhyay et al. 2020), and the discharges of untreated sewage and industrial wastewater (Nethaji Mariappan et al. 2017). Also in the Buckingham Canal, that receives sewage and industrial effluents during its course through the city and the silting up of the canal has left the water stagnant (Jayaprakash et al. 2012). Encroachment in some sections of the central area has shrunk its width from 100 m to 10 m (2019b).

The lakes and the Pallikaranai marshland have suffered from encroachments, dumping, burning of wastes and sewage, and industrial wastewater discharge (Shahina et al. 2020). The Pallikaranai marshland is additionally polluted by the non-isolated Perungudi dumpsite (Sree Sharmila and Swathika 2016). High concentrations of Cd, Hg, Cr, Cu, Ni, Pb, and Zn were found in its sediments (Jayaprakash et al. 2010). Seawater has intruded up to 16.5 km inland in the Minjur - Panjetty area due to over-exploitation of groundwater (Government of India 2017).

Seawater is polluted nearby the river mouths of the Adyar and the Cooum rivers by high concentrations of nutrients, low dissolved oxygen, high biochemical oxygen demand (BOD) and chemical oxygen demand (COD), high total suspended matter, high trace elements (Shanmugam et al. 2007; Mishra et al. 2015), high level of coliforms, Vibrio and Pseudomonas, (Santhiya et al. 2011), and presence of antibiotic-resistant pathogens (Vignesh et al. 2012) and microplastics (Kumar et al. 2016).

#### Reduction of Biodiversity and Human Health

Amali et al. (2019) found that the wetlands located within the coastal districts of Tiruvallur, Chennai, and Kancheepuram have decreased to 31.6%, from 23.1% in 1988 to 15.8% in 2016, i.e., an average annual reduction of 1.35%.

The degradation of aquatic ecosystems in Chennai has impacted its biodiversity. Invasive species, like the ornamental fish suckermouth, have been introduced and affected the local species population (Oppili 2017). Water hyacinth colonized and significantly affected the biodiversity in Pallikaranai Marshland (Surya 2016). The construction of roads fragmented the marshland and limited the movement of animals (Azeez et al. 2007), with a reduction of resident birds in general (Vencatesan 2007) and the Black-winged Stilt in particular from 836 individuals in 2000 to 150 in 2010 (Raj et al. 2010).

For Buckingham Canal, the changes in the water quality reduced the primary productivity, affecting the entire aquatic ecosystem (Kumar et al. 2018). In the Ennore estuary, 15 species were found dead in large numbers in August 2014 (Sachithanandam et al. 2017). High concentrations of Fe, Mn, Zn, Cu, Pb, and Cd in *Mugil cephalus* fish and ultrastructural alterations on the green mussel *Perna viridis* have been found (Arockia Vasanthi et al. 2013; Vasanthi et al. 2017). At the discharge site of the power plant located in Ennore, the population density of phytoplankton and zooplankton is reduced by 64% and 93%, respectively (Prince Prakash Jebakumar et al. 2018).

The pollution of water resources has also adversely affected Chennai’s inhabitants health. The stagnant water in the Buckingham Canal creates an attractive habitat for malaria-spreading mosquitoes (Jayaprakash et al. 2012). Water-borne diseases like Diarrhea and Typhoid are common (Sethuram 2014). Drinking water in some areas was highly contaminated with coliforms, *Cryptosporidium,* and *Isospora* (Anbazhagi et al. 2007; Lavanya 2013; Chopra and Dworkin 2013), including sachet water but not bottled water (Venkatesan et al. 2014).

### Responses

#### Urban Planning

Chennai has had two master plans for urban planning. The First Master Plan for the Chennai Metropolitan Area was approved in 1976 and the Second is in force since September 2008 (CMDA 2020). Nevertheless, the urban form of the Chennai Metropolitan Area has been dictated by developments along the major roads and rail links radiating from the center of Chennai into the peri-urban regions (CMDA 2008). The second Plan included chapters for infrastructure for water supply and sanitation, with a recommendation to allocate water to areas within the city and outside, and for the environment, with a recommendation to reduce pollution levels to acceptable standards in the waterways of Chennai. Recently, the elaboration of a third master plan with long-term vision document produced through a participatory approach has been announced, being planned to be implemented from 2026 and to last for two decades (Shivakumar 2020).

#### Water Infrastructure

Chennai has developed a big water infrastructure consisting on dams and interbasin transfers, groundwater pumping, rainwater harvesting, desalination plants, and water tankers.

Over 90% of the water supply of Chennai is covered by water stemming from reservoirs (Brunner et al. 2014; Sakthivel et al. 2015). Surface water is provided mostly through two systems of interconnected reservoirs. The oldest system has three reservoirs at Poondi (91.5 hm^3^) and Cholavaram (25 hm^3^), that discharge into Puzhal (93.5 hm^3^), also called Red Hills. This water is treated at Puzhal (300 million liters per day, MLD), Kilpauk (270 MLD), and Surapet (14 MLD) (CMDA 2006; CMWSSB 2020b). The newest system has Chembarambakkam reservoir (103.2 hm^3^) and Chembarambakkam Water Treatment Plant (530 MLD) (CMDA 2006; CMWSSB 2020b).

When water is scarce, around 500 MLD water from the Srisailam reservoir in the Krishna River, can be diverted towards Poondi Lake through a series of 400 km-long interlinked canals called the Telugu Ganga project (CMDA 2006). Also, Veeranam Lake and Vadakuthu Water Treatment Plant (180 MLD) are added to the system. In total, the capacity of the cited water treatment plants is 1294 MLD (CMWSSB 2020c).

Groundwater’s contribution has significantly diminished due to over-exploitation (Packialakshmibb et al. 2011; Sakthivel et al. 2015) from a maximum of 25% to around 6% during the 2000’s (Brunner et al. 2014). Because of the low water level in the inner-city, Metrowater started to hire private peri-urban agricultural wells in 2000 that yielded 77 MLD in 2005 compared to only 5.99 MLD of previous wells (CMDA 2006). Currently, Metrowater continues extracting in peri-urban areas: Minjur, Panjetty, Tamaraipakkam Poondi, and Kannigaiper (CMWSSB 2020d). In fact, most of the agricultural wells in the villages in the Chennai Metropolitan Area feed the inner-city water market through water tankers (Packialakshmibb et al. 2011) depriving peri-urban farmers of irrigation water (Butterworth et al. 2007). In the southern peri-urban interface, 17.1 MLD are extracted by private water tankers and 19.2 MLD by packaged water industries (Packialakshmibb et al. 2011). Also many households in Chennai have in-house wells (Ruet et al. 2007).

To improve this situation, installation of Rain Water Harvesting (RWH) structures became mandatory for new buildings in 2002 (CMWSSB 2020d). Since then, 38,218 structures have been constructed (CMWSSB 2020d) with high acceptance by the public (Vivek 2016) but maintenance issues were reported (Ministry of Water Resources, Government of India 2011).

Since 2010, a 100 MLD seawater desalination plant is operating at Minjur, north of the city, and a similar 100 MLD plant at Nemmeli, south of the city, since 2013 (CMWSSB 2020e). A third 150 MLD plant is expected to be completed by April 2023 (Lakshmi 2022) and a fourth 400 MLD at Perur by 2024 (Lakshmi 2019).

Tanker-based water supply is estimated to be 21% of the total water supplied in Chennai (Maheshwari et al. 2020) i.e. around 125 MLD (Venkatachalam 2015), that would be delivered by up to 20,000 daily tanker loads of water (The Hindu 2018). About 80% of households in the Chennai Metropolitan Area consume packaged drinking water instead of piped water (The Hindu 2018). About 15–20 MLD of packaged drinking water are sold in the city daily (Srinivasan 2008).

#### Sewage Treatment

The total installed wastewater treatment capacity in Chennai reaches 727 MLD in 12 sewage treatment plants, however, it is estimated that nearly 1073 MLD of sewage are directly discharged into water bodies (Goutham 2017). The 12 treatment plants are divided in zones from I to V and their treatment capacity ranges from 12 to 120 MLD. In Nesapakkam, Koyambedu, and Kodungaiyur, plants use activated sludge process and in Villivakkam an aerated lagoon (CMDA 2020). In 2005 and 2006, the Chennai City River Conservation Project (CCRCP) invested Rs. 7201.5 million to increase the sewage treatment capacity, prevent overflow of sewage into the city waterways, and renovate part of the system (CMDA 2006).

#### Restoration of Water Bodies

There are several projects to restore Chennai’s water bodies. The Chennai Rivers Restoration Trust, founded in 2006 by the Government of Tamil Nadu as Adyar Poonga Trust, has three projects: the Eco-Restoration of Adyar Creek (58 acres), the Eco-Restoration of the Adyar Creek and Estuary (300 acres) and the Integrated Cooum River Eco-Restoration Plan (Chennai Rivers Restoration Trust 2020).

The non-governmental organizations (NGOs) Care Earth Trust and The Nature Conservancy, among others, have developed wetland restoration projects introducing a sustainability and community participation focus. Care Earth Trust has successfully completed the restoration of the Narayanapuram Lake, the Perungalathur Periya Eri, the Puduthangal Lake, Thazhambur Eri, the Sembakkam Lake, the Puducheri Keni kulam and the Odai keni kulam (Care Earth Trust 2020).

In the Pallikaranai marshland, 317 ha of its southernmost portion were declared as a bird sanctuary on April 2007 (Vencatesan 2007; Steinbruch and Hörmann 2015) and its declaration as Ramsar site is in progress (Shekhar 2020).

#### Water Governance

The Chennai Metropolitan Water Supply and Sewerage Act, 1978, amended in 1997, also known as Tamil Nadu Act 28 of 1978 founded Metrowater and gave it the mission to provide the Chennai Metropolitan Area an adequate supply of good quality water and a safe disposal of wastewater at a reasonable price (CMWSSB [Bibr CR42][Bibr CR42]). Metrowater subsidizes its residential customers by overpricing its industries (Gopakumar 2009). According to their data, 830 million liters per day (MLD) of drinking water are produced in Chennai, whereas the treatment capacity reaches 1494 MLD. The expansion to cover the whole Chennai Metropolitan Area is in progress (CMWSSB 2020c). A few years later, the Chennai Metropolitan Area Ground Water (Regulation) Act, 1987 came into force to preserve groundwater and gave Metrowater the power to authorize permits to sink wells and licenses for extraction and the obligation of keeping well registers and the use of groundwater for agricultural purposes (The Chennai Metropolitan Area Groundwater (Regulation) act 1987). Furthermore, The Tamil Nadu Pollution Control Board is responsible for setting, monitoring, and enforcement of environmental regulations and standards (Roumeau et al. 2015).

Current approaches to water governance struggle to meet Chennai’s demands for water. Legislation exists but for political, institutional, and social reasons it is not always enforced (Janakarajan and Lakshmi 2005).

The insufficient supply of water by public entities has made private owned tankers and packaged water vendors critical actors in water governance (Roumeau et al. 2015). Many farmers have left their agricultural activities to sell groundwater directly to private tankers following a greater revenue (Packialakshmibb et al. 2011; Roumeau et al. 2015). Other stakeholders are water suppliers in the private sector, farmers, and civil society (Roumeau et al. 2015) as well as multiple NGOs dealing with water and environmental issues, like the Environmentalist Foundation of India or the Care Earth Trust.

## Discussion

Different studies have assessed the DPSIR framework in terms of its application for sustainable development studies (Carr et al. 2009), determination of causal relationships (Niemeijer and De Groot 2008), and its feasibility for supporting decision-making (Tscherning et al. 2012). These authors enunciate that the DPSIR presents drawbacks regarding to its hierarchical structure, the establishment of unidirectional relationships, and, in some studies, the lack of integration of decision-makers into the participative process. Nevertheless, they also stress the DPSIR’s potential to emphasize causality between environmental indicators and to structure disperse data to provide meaningful explanations of complex environmental issues to policymakers (Niemeijer and De Groot 2008; Tscherning et al. 2012) that facilitates communication and comparison (Patrício et al. 2016). Since the development of the DPSIR, 25 derivatives in more than 152 studies have been proposed in the literature (Taft and Evers 2016; Patrício et al. 2016). However, none has been adopted by the scientific community as a replacement for the DPSIR nor by international institutions as the original DPSIR framework was by the European Union and European Environment Agency (European Commission 1999).

Through the application of the DPSIR in Chennai, the authors agree on its advantages and its potential to be connected with SDGs 6 and 11. The application of the DPSIR involves indicators regarding water resources and pollution, waste, land use change, and loss of biodiversity among others (Smeets and Weterings 1999; Kristensen 2003; European Environment Agency 2005, 2014) that are also included in the SDGs 6 and 11, like the indicators 6.3.1 Proportion of domestic and industrial wastewater flows safely treated, 6.3.2 Proportion of bodies of water with good ambient water quality or 6.6.1 Change in the extent of water-related ecosystems over time and 11.3.1 Ratio of land consumption rate to population growth rate (United Nations 2022a, b).

The application of the DPSIR framework also found limitations on water management. First, water management requires to be faced from several perspectives and considering a large number of variables. Today, decision-makers and stakeholders work in many cases independently with contradictory interests. Therefore, a deeper integration of the public administration bodies involved in water management and better cooperation of different stakeholders is necessary. Secondly, poor law enforcement hinders efficient water management. For example, groundwater over-extraction in peri-urban areas. Thirdly, data scarcity or difficult access impedes to develop new efficient policies adapted to the city needs.

In the Indian context, the implementation of effective policies to manage water challenges in cities is limited by inaccessible and unreliable data, limited coordination between stakeholders, non-implementation of policies and projects, environmental legislation violations, unclear or overlapped responsibilities, inadequate maintenance of water infrastructure (Aartsen et al. 2018). The Organisation for Economic Cooperation and Development (OECD 2015) provides a framework to understand water governance systems’ performance. In addition, the implementation of adaptive management approaches has been stated as a comprehensive solution for improving decision-making and managing water resources (Pahl-Wostl 2008; Kattel 2019a). For instance, Kattel, 2019a, 2019b, has proposed six key elements for enhancing water security in river basins i.e. the implementation of early warning systems, water footprints management, groundwater aquifers management, increased water stewardship and governance, enhanced resilience of social and ecological systems of freshwaters and the promotion of the water-energy-food nexus.

Chennai and its metropolitan area are expected to keep facing water stress and difficulties to implement the SDGs 6 and 11. The current water supply system has proven to be insufficient even with average weather conditions, with a gap of 342 MLD between estimated demand (1173 MLD) and supply (831 MLD) in 2014 (Government of Tamil Nadu 2014) and with a supply of only around 90 liters per capita a day (CMDA 2006). Chennai needs to urgently find new water sources to meet the always-increasing demand.

Conventional surface water sources, like new reservoirs, are fed by the Northeast Monsoon and would face a similar trend of drought (Chennai Metropolitan Development Authority, 2006). Catchments depending on the Southwest Monsoon are far from Chennai and would require high costs to build and operate long channels and tensions between communities would emerge, like in the Telugu Ganga project. Groundwater is unable to supply sufficient water in a sustainable manner. Recharging the aquifers by water harvesting would contribute to a more stable and reliable water supply. The two new seawater desalination plants (2 × 500 MLD) planned for 2024 can cater more than 2 million people, although a final affordable water price has to be granted for poorer areas. Otherwise, water bills could remain unpaid as it happens in the rest of the state of Tamil Nadu (Gopakumar 2009). Furthermore, the management of brine produced by desalination plants requires to be considered.

Chennai’s water bodies are being polluted because of the lack of wastewater treatment plants. The likely 1073 million liters per day (MLD) gap between treated (550 MLD) and non-treated (1500–2000 MLD) sewage is directly discharged into water bodies (Goutham 2017; CMWSSB 2020a). However, it is insufficient to cover the present and future sewage generation. The authorities need to find funds to build new sewage treatment plants and an efficient waste management system, including the closure of the current two main open dumpsites. The ability of the institutions to implement urban plans that consider sewage treatment and waste management systems will be key for pollution prevention of water bodies at a lower cost. The third master plan of Chennai brings a new and promising focus with a long-term vision document produced through a participatory approach.

Biodiversity in the Pallikaranai marshland and other water bodies has been reduced due to increased water pollution and invasive species like the water hyacinth (Oppili 2017). NGOs like Care Earth Trust, The Nature Conservancy, and the Environmentalist Foundation of India play a major role in the restoration of aquatic ecosystems. Restoration success relies significantly on the interest of the institutions to implement this kind of projects on their own or with the help of NGOs and also in the ability of these NGOs to achieve external funds.

Environmental regulations need to meet international standards and to be enforced to ensure the protection of the aquatic ecosystems (Janakarajan and Lakshmi 2005). In May 2020, a new draft of the Environment Impact Assessment Notification 2020, under the Environment (Protection) Act, 1986 came into force. It set aside several essential provisions such as public consultation and introduced ex-post facto clearance for many projects, including some irrigation and hydroelectric projects, all inland waterway projects, and others (Ananthakrishnan 2020). This sets a worrying precedent that could affect other environmental regulations.

The severe hit of the COVID pandemic on the Indian economy dropped investment growth in the year 2020 to 10% (United Nations 2021) and this will probably delay many of the mentioned projects.

## Conclusions

This study applied the DPSIR Framework to water management in Chennai, South India. The study provides answers to the research questions raised in the introduction section. First, the DPSIR proved to be a valuable tool to evaluate water challenges in cities by disentangling relationships between environmental indicators and structuring dispersed data that allows policymakers for a better understanding. The indicators used for the DPSIR framework can be also easily aligned with the indicators of the Sustainable Development Goals 6 and 11. Second, limited integration of decision-makers and stakeholders, poor law enforcement, and scarcity of data limit effective water management in Chennai, which can be applied to other cities with similar scenarios.

As a result of the DPSIR analysis, the unplanned population growth fueled by rapid economic development was identified as the two main drivers. They brought pressures such as rapid land use change, groundwater over-extraction to partially cover the higher water demand, and bigger volumes of solid wastes and untreated sewage that are discharged into water bodies. This situation headed to low values of indicators related to the status of urbanization, water availability, water quality and biodiversity, and visible environmental impacts within the city: loss of aquatic ecosystems and encroachment, low water table levels, low water quality, that reduces aquatic biodiversity and human health. Urban planning and the third Chennai master plan were identified as a key response. Stakeholders executed numerous water infrastructure projects that increased drinking water availability with conventional (dams and interbasin transfers, groundwater pumping) and non-conventional (desalination plants, water tankers, rainwater harvesting) techniques. They also built sewage treatment plants, restored ecosystems, and improved water governance.

## Supplementary Information

Supplementary Information

## Data Availability

The datasets generated during and/or analysed during the current study are available from the corresponding author on reasonable request.
