# Supplementary material for: Rethinking Urban Water Management Through Drivers-Pressures-States-Impacts-Responses Framework Application in Chennai, India
Source: Environ Manage. 2024 Aug 6;74(5):970–88. doi: 10.1007/s00267-024-02022-z (PMC11438619; doi:10.1007/s00267-024-02022-z)
Supplement: Supplementary file 1 — Supplementary Information [file 267_2024_2022_MOESM1_ESM.docx]

# Supplementary material

Table S1. Main indicators considered for every component of the Drivers-Pressures-States-Impacts-Responses analysis of water management in Chennai in this study.

|  |  | Indicator | Value | Years | Source |
| --- | --- | --- | --- | --- | --- |
| Drivers | Population growth | Average annual population growth (%) | 0.75 | 2001-2011 | Directorate of Census Operations Tamil Nadu (2011) |
|  | Economic development | Average annual growth of per capita net state domestic product in rupees at constant prices in Tamil Nadu (%) | 6.59 | 1998-1999 to  2018-2019 | Reserve Bank of India (2020) |
| Pressures | Land use change | Average annual increase of urban area (%) | 1.92 | 1988-2017 | Mathan and Krishnaveni (2020) |
|  | Groundwater over-extraction | Average groundwater extracted (million liters per day) | 200 | 2015 | Venkatachalam (2015) |
|  | Liquid and solid waste mismanagement | Waste water treated (%) | 33.90 | 2017 | Arappor Iyakkam (2017) |
| States | Urbanization status | Urban area over the total of the Chennai Metropolitan Area (%) | 48.7 | 2017 | Mathan and Krishnaveni (2020) |
|  | Water availability | Water supply (liter/inhabitant/day) | 90 | 2014 | Government of Tamil Nadu (2014) |
|  | Water quality | Biochemical oxygen demand (mg oxygen/l) | 9-375 | 2015-2019 | Kumar et al. (2019); Dhamodharan et al. (2016); Nethaji Mariappan et al. (2017); Kumar et al. (2018); Raji and Abraham (2018); Krishna Kumar et al. (2015) |
|  | Biodiversity status | Wetland area over the total of the Chennai Metropolitan Area (%) | 15.80 | 2016 | Amali et al. (2019) |
| Impacts | Loss of aquatic ecosystems | Average annual reduction in water surface (%) | 1.25 | 1988-2016 | Mathan and Krishnaveni (2020) |
|  | Low water table | Water extracted of the aquifer (%) | 80 | 2012 | Balan et al. (2012) |
|  | Low water quality | Untreated waste water discharged (million liters per day) | 1073 | 2017 | Arappor Iyakkam (2017) |
|  | Reduction of biodiversity and human health | Average annual reduction in wetlands (%) | 1.35 | 1988-2016 | Amali et al. (2019) |
